# Virtual Reality for Patient Education about Hypertension: A Randomized Pilot Study

**DOI:** 10.3390/jcdd10120481

**Published:** 2023-11-29

**Authors:** Bogna Jiravska Godula, Otakar Jiravsky, Gabriela Matheislova, Veronika Kuriskova, Alena Valkova, Kristina Puskasova, Martin Dokoupil, Veronika Dvorakova, Arber Prifti, Daniel Foral, Filip Jiravsky, Jan Hecko, Miroslav Hudec, Radek Neuwirth, Roman Miklik

**Affiliations:** 1 Department of Cardiology, Agel Hospital Trinec-Podlesi, 739 61 Trinec, Czech Republicjan.hecko@npo.agel.cz (J.H.);; 2 Poliklinika Agel Ostrava, Dopravni Zdravotnictvi, 728 06 Moravian Ostrava, Czech Republic; 3 Faculty of Medicine, Palacky University, 779 00 Olomouc, Czech Republic; 4 Faculty of Medicine, Masaryk University, 625 00 Brno, Czech Republic; 5 Agel Hospital Ostrava Vitkovice, 703 00 Ostrava-Vítkovice, Czech Republic; 6 Philosophical Faculty, Masaryk University, 602 00 Brno, Czech Republic; 7 Faculty of Electrical Engineering and Computer Science, VSB-Technical University of Ostrava, 708 33 Ostrava, Czech Republic

**Keywords:** virtual reality, patient education, hypertension, knowledge, randomized controlled trial

## Abstract

Background: Hypertension challenges arise in part from poor adherence due to inadequate patient education. VR offers immersive learning to improve hypertension knowledge. Objective: To compare VR education with traditional verbal education to improve hypertension knowledge. Methods: In this randomised trial, 182 patients with hypertension were assigned to receive either traditional physician-led education (n = 88) or VR education (n = 94) with equivalent content. The VR group experienced a 3D video using Oculus Quest 2 headsets. Knowledge was assessed post-intervention using a 29-item questionnaire. The primary outcome was the objective score. Subjective satisfaction and responder characteristics were secondary outcomes. Results: Median objective scores were significantly higher for VR (14, IQR 3) versus traditional education (10, IQR 5), *p* < 0.001, indicating superior hypertension knowledge acquisition with VR. Subjective satisfaction was high in both groups. Participants were categorized into low (first quartile) and medium-high (second to fourth quartiles) responders based on their scores. Low responders had a significantly higher prevalence of older women than medium-high responders (57% vs. 40% female, *p* = 0.024; 68 vs. 65 years), *p* = 0.036). Conclusions: VR outperforms traditional education. Tailoring to groups such as older women can optimise learning.

## 1. Introduction

Hypertension remains a major global public health challenge, affecting more than 1.4 billion adults worldwide [1,2]. Suboptimal blood pressure control rates persist, with only about 40% of patients with hypertension achieving recommended targets worldwide [3]. Poor adherence to pharmacological therapy and lifestyle modifications are major contributing factors [4,5]. However, improving patient understanding of hypertension through education is critical to facilitate adherence and behavioral changes to optimise management [4,6,7].

Traditional physician-led methods such as face-to-face verbal explanations, booklets, group classes, and other standard approaches have proven beneficial in delivering hypertension education [7,8,9]. However, innovative technologies such as virtual reality (VR) are changing the way patients learn about medical conditions and treatments [10,11,12]. VR refers to computer-generated simulations of 3D environments that users can interact with and immerse themselves in through headsets and sensory feedback [10,13]. VR is proliferating in healthcare, with demonstrated efficacy for anatomical and surgical education [14,15,16], as well as applications in surgical training, rehabilitation, psychotherapy, and more [17,18,19].

In patient education, VR creates immersive, interactive experiences that engage users more deeply than passive techniques [10,12]. VR environments have expanded the ability to tailor learning to individual needs and make abstract concepts more understandable [10,19]. Similarly, Augmented Reality (AR) offers a highly realistic learning experience by augmenting real-world perceptions with virtual content to support the mastery of complex skills in medical learning, which is particularly relevant where training in real-world contexts is limited by safety, cost or didactic constraints [20]. However, significant knowledge gaps remain regarding the usefulness and acceptability of VR for patient education compared to conventional teaching [21,22].

In the context of outpatient clinics, the potential for VR to complement traditional educational methods remains an area ripe for exploration. Although traditional educational strategies are effective, they may not fully address the diverse learning needs of patients. This study evaluates the use of VR in this setting and seeks to understand its utility in improving educational outcomes for physicians treating hypertension. By integrating VR into physician-led educational strategies, we anticipate that the immersive and interactive nature of VR can provide an enriched learning environment that may improve patient understanding and management of hypertension.

This randomised controlled trial addresses this gap by comparing VR-based education to traditional physician-led verbal education for improving patient knowledge of hypertension. We hypothesised that the VR modality would be noninferior or superior to standard education for knowledge acquisition.

## 2. Methods

### 2.1. Participants and Study Design

This was a prospective, randomised, single-blind controlled, parallel-group study (registered with ClinicalTrials.gov (NCT05735808)). Participants were recruited from the patient population of internal medicine and general practice clinics. Patients were eligible if they were 18 years of age or older and had a diagnosis of essential hypertension managed on an outpatient basis. Exclusion criteria were significant cognitive impairment precluding informed consent or questionnaire completion, significant vision loss or physical disability preventing proper use of the VR headset, or previous formal education on hypertension resulting in advanced prior knowledge.

### 2.2. Randomisation and Blinding

Eligible, consenting participants were randomly assigned in a 1:1 ratio to receive education using either the traditional physician-led verbal method (control) or the VR method (intervention). Randomisation was performed in blocks of 20 using computer-generated sequences by an independent statistician. Allocation concealment was achieved using sequentially numbered opaque sealed envelopes. The clinicians providing the interventions and the analysts were blinded to the group assignments. Participants were blinded to the study hypotheses but not to the intervention they received.

### 2.3. Interventions

#### 2.3.1. Development and Validation of Educational Content

Equivalent educational content on key hypertension basics was developed for both groups in collaboration with hypertension specialists at the study site. The learning objectives were to improve understanding of (1) the definition of hypertension, (2) risk factors, (3) common symptoms, (4) appropriate use of medication, (5) home blood pressure monitoring;,and (6) potential complications.

For the VR intervention group, this content was adapted into an eight-minute 3D educational video filmed with a 360-degree camera (Insta360 ONE X2; Insta360, Shenzhen, China) at one of the study clinic sites. The video was enhanced with diagrams, animations, and a voiceover script to simulate a typical doctor’s visit. It was then imported into Oculus Quest 2 (Reality Labs, Meta Platforms, Menlo Park, CA, USA) standalone wireless VR headsets.

Participants who were randomised to the VR group, underwent a single education session in which they experienced immersive learning by watching the 3D video in the VR headset. They were free to move around the virtual environment, which was modelled on the clinic rooms.

For the control group, participants received traditional standardised face-to-face verbal education sessions delivered by trained study doctors. These sessions were equivalent in content to the VR video, matched in duration, and used a slide deck with diagrams.

The VR and traditional education sessions were conducted face-to-face in quiet clinic rooms at the study site. Participants could ask questions, and clinicians repeated explanations as needed.

In terms of the cost and logistics of the educational methods used in this study, five Oculus Quest 2 VR headsets were purchased at a cost of €400 each, and an Insta360 ONE X2 camera was purchased at a cost of €350 to create the VR content. The development of the learning materials and the filming and post-production processes were carried out in-house at no additional cost. Each VR training session was standardised to last 8 min, followed by a question and answer period to ensure interactivity and engagement. Similarly, the control group received face-to-face training of the same length, with an equivalent opportunity to ask questions. This structure ensured that both the VR and traditional training methods were comparable in terms of delivery time and interaction potential, with costs contained through efficient use of resources and self-production of educational content.

During the preparatory part of the study, a rigorous review process was undertaken to ensure the validity of the educational content. Materials for both VR and traditional education were developed in collaboration with hypertension specialists and validated by peer review to ensure accuracy and relevance.

All educational materials and sessions, for both the VR and traditional face-to-face methods, were conducted in Czech to ensure full comprehension and comfort for our native Czech-speaking patient cohort. This consideration was crucial to maintain consistency in the delivery of information and to facilitate effective communication during the educational interventions.

#### 2.3.2. Validation of Audiovisual and VR Content

Audiovisual elements for the VR intervention, including diagrams and animations, were critically reviewed by a panel of experts in medical education and revised according to their feedback to ensure that the content was pedagogically sound.

#### 2.3.3. VR Headset Testing and Reliability

The Oculus Quest 2 headsets were tested for reliability prior to the start of the study. This included checking for software stability, battery life, and user comfort during prolonged use. Any headsets that did not meet the performance criteria were excluded from the study.

#### 2.3.4. Standardisation of Face-to-Face Training

To ensure consistency in the delivery of face-to-face education, all participating clinicians underwent standardised training. This training included calibration sessions to align teaching styles and ensure consistent delivery of content.

#### 2.3.5. Interactivity of Educational Sessions

Both educational approaches were designed to be interactive. Participants in both groups had the opportunity to ask questions and facilitators were instructed to provide comprehensive answers. The Q&A sessions were of equal duration and structured to match the interactivity potential of the VR sessions.

#### 2.3.6. Opportunity to Post-Trial Experience

Following completion of the randomised education sessions and subsequent data retention checks, participants were informed that they had the opportunity to experience the alternative education method. This was offered for experiential purposes only and did not contribute to the study data. The VR group was able to engage with the traditional method and vice versa to allow participants to fully appreciate the different educational modalities. This aspect of the study was conducted after the primary outcome measures had been assessed to avoid any influence on the efficacy data of the assigned interventions.

### 2.4. Outcomes

#### 2.4.1. Knowledge

The primary outcome was objective knowledge of hypertension, assessed immediately after the education. A 29-item questionnaire covering the six learning objectives was developed by the study investigators, with 4–5 multiple-choice questions per topic. Content validity was assessed by having three hypertension specialists review the questionnaire and confirm its appropriateness for measuring the knowledge concepts of interest.

To ensure fair assessment, the test items were carefully designed to reflect the content delivered during both the VR and traditional physician-led training sessions. Each question was directly related to the information presented, ensuring that no specific prior knowledge of the topics was required to answer correctly. This comprehensive approach allowed us to accurately measure the effectiveness of the educational interventions, regardless of the participants’ initial level of knowledge. The distribution of test items across the objectives was designed to assess all areas covered equally, with the presentation of results tailored to show a holistic view of knowledge retention across all topics.

The questionnaire was piloted on a small group of patients for feedback before finalisation to control readability. Item analysis was used to remove poorly performing questions. The total objective score was calculated by assigning (+1) point for each correct answer and (−1) point for each incorrect answer, with a maximum score of 29.

#### 2.4.2. Subjective Satisfaction

Subjective satisfaction with the educational modality was assessed as a secondary outcome. To capture this, we used a 5-point Likert scale, with 1 representing ‘extremely satisfied’ and 5 representing ‘extremely dissatisfied’. This scale was chosen specifically because of its ubiquity in the Czech education system, making it a familiar method of assessment for all study participants, thus reducing potential biases in the interpretation of satisfaction levels.

The survey instrument was designed taking into account the cultural context and the consistency with which satisfaction measures are traditionally reported in Czech education. Its reliability and validity are supported by its longstanding use in academic settings in the country, and it has been shown to effectively discriminate between different levels of satisfaction in the Czech-speaking population.

Mean scores from the satisfaction survey were interpreted in accordance with conventional practice: lower scores indicate higher satisfaction and higher scores indicate lower satisfaction. The means were also compared between the two groups to see if there was a statistically significant difference in satisfaction with the educational methods.

#### 2.4.3. Responder Analysis

First, the distribution of total objective scores was examined by calculating standard deviations and interquartile ranges separately for the VR group and the physician-led group.

Levene’s test for equality of variances was then used to statistically compare the homogeneity of variance in scores between the two education groups. This analysis was designed to assess the consistency of results between the VR and physician-led methods.

Next, participants in each education group were separately categorised into “low responders” (those scoring in the first quartile Q1) and “medium-high responders” (those scoring in the second to fourth quartiles Q2–Q4) based on their total objective scores.

Finally, the characteristics of low versus medium-high responders within each education group were compared using Pearson’s chi-squared and Mann–Whitney U tests. This analysis was designed to identify factors potentially associated with lower scores, including demographics such as age and gender.

#### 2.4.4. Adverse Effects

In our study, we carefully considered the potential adverse effects associated with VR training. In the consent form, participants were informed of the possibility of feeling disorientated, particularly those prone to dizziness. To address these concerns, we advised participants to remain seated during and immediately after the VR session. In addition, participants were instructed not to stand up without the assistance of a healthcare professional. These precautions were taken to mitigate any temporary adverse effects of VR use and were clearly communicated to participants prior to the start of the study.

### 2.5. Statistical Analysis

Intention-to-treat analysis was performed using IBM^®^ SPSS^®^ Statistics software, version 28.0.0.0. We classified the variables measured in our study as follows: continuous variables such as age and knowledge assessment scores, which were presented as mean (±standard deviation) for normally distributed data and median (interquartile range) for non-normally distributed data. Categorical variables included demographic information such as gender, comorbidities and responses to the satisfaction survey, reported as frequencies (percentages).

The Shapiro–Wilk test was used to assess the normality of continuous variables. The Mann–Whitney U test was used to compare knowledge scores between the VR and traditional education groups when the distribution was not normal. Pearson’s chi-squared or Fisher’s exact tests were used to analyse categorical variables.

Levene’s test for equality of variances was used to assess the consistency of results, and all analyses were performed at a significance level of *p* < 0.05.

## 3. Results

### 3.1. Participant Flow and Baseline Data

Of the 200 patients initially screened, 182 diagnosed with arterial hypertension were deemed eligible and enrolled in the randomised controlled trial. These participants were then randomly assigned to receive either traditional, physician-led education (n = 88) or virtual reality education (n = 94). Notably, no dropouts or withdrawals were reported during the course of the study. All randomised participants completed the educational intervention and post-education assessments as per protocol, providing complete data for the final analyses.

Baseline characteristics were balanced between groups after successful randomisation (Table 1). The overall cohort had a median age of 66 (IQR 16) years and 54% were male. The median age was similar in the VR (66 years) and physician-led education (66 years) arms, as was the gender distribution (55% and 54% male, respectively). Other baseline parameters, including blood pressure, antihypertensive medication use, hypertension control rates, and comorbidities, were not significantly different between groups (all *p* > 0.05), confirming successful randomisation.

### 3.2. Results-Knowledge

For the primary outcome of median objective knowledge scores, the VR education group demonstrated clear superiority over the conventional physician-led education group (Table 2). The median score achieved with VR education was 14 (IQR 3) compared with 10 (IQR 5) for conventional (physician-led) education. This 4-point difference was statistically significant (*p* < 0.001), demonstrating greater hypertension knowledge acquisition with the VR modality.

#### Test of Difference

Next, the distribution of knowledge measured by the total objective scores was examined by calculating standard deviations and interquartile ranges separately for the VR group (SD 2.8, IQR 3.0) and the physician-led group (SD 3.5, IQR 5.5) (Supplementary Table S1). Levene’s test for equality of variances was then used to statistically compare the homogeneity of variance in scores between the two education groups. This analysis showed significantly greater heterogeneity in the physician-led group compared to the VR group (*p* = 0.003 to *p* = 0.015 using mean, median, adjusted median, and trimmed mean) (Supplementary Table S2).

### 3.3. Results-Satisfaction

Both education methods received high subjective satisfaction ratings (Table 3). Overall, 84% of participants reported the maximum satisfaction score of 1 on the 5-point Likert scale. The majority in both the VR (88%) and conventional education (81%) groups were “very satisfied” with their assigned intervention. Median satisfaction scores for the VR and conventional arms were 1 (IQR 0) and 1 (IQR 1), respectively, with no significant differences between groups (*p* = 0.246).

### 3.4. Responder Analysis

Participants in each education group were separately categorised into “low responders” (those scoring in the first quartile; Q1) and “medium-high responders” (those scoring in the second to fourth quartiles; Q2–Q4) based on their total objective scores.

The characteristics of low versus medium-high responders were then compared within each education group. This analysis revealed a significantly higher prevalence of older women in the low responder subgroup, as evidenced by differences in gender (57% vs. 40% female, *p* = 0.024) and age (68 vs. 65 years, *p* = 0.036) (Table 4).

However, subjective satisfaction was not significantly different between low and medium-high responders (median score 1 for both, *p* = 0.295). This suggests that participants’ perceived satisfaction does not necessarily correlate with objective knowledge retention.

In summary, the responder analysis revealed more heterogeneous, inconsistent results in the physician-led group compared to the VR education group. It also identified older female gender as potentially associated with lower objective scores, but not subjective satisfaction.

### 3.5. Subanalysis of Knowledge

A subanalysis was performed to examine the differences in objective knowledge gains between the VR and physician-led education groups for each specific question topic (Supplementary Table S3).

In the Definition section, there were no significant differences between groups, indicating comparable performance.

However, in the Risk Factors and Symptoms sections, the VR group had significantly fewer incorrect answers on nearly all questions (*p* < 0.05). For example, only 3.2% of the VR group incorrectly identified family history as a risk factor, compared with 34.1% of the physician-led education group (*p* < 0.001), while only 7.4% of the VR group incorrectly identified chest pain as a symptom, versus 44.3% of the physician group (*p* < 0.001).

In the Regular Medication Use section, there were no significant differences. However, in the Home Blood Pressure Measurement and Complications sections, the VR group again demonstrated fewer incorrect responses on most questions (*p* < 0.05). For instance, only 11.7% of the VR group incorrectly identified how often to measure blood pressure at home, compared to 43.2% of the physician group (*p* < 0.001).

In summary, this subanalysis by question topic identified specific knowledge areas where VR education appeared to be beneficial, including identification of risk factors and symptoms of hypertension, proper home monitoring, and awareness of complications. The more consistent and significantly better performance of the VR group in many specific content areas provides further evidence that this novel modality may confer benefits in improving hypertension understanding compared to standard education. The differences between the groups in the proportion of incorrect answers to certain questions indicate areas where the educational content in both modalities could be improved to further optimise learning. Tailoring the material to address the gaps highlighted by this subanalysis may help standardise knowledge acquisition across question topics for both VR and traditional (physician-led) education.

### 3.6. Adverse Events

Reassuringly, there were no reported adverse events or complications associated with either education method throughout the duration of this randomised controlled trial. All participants successfully completed the study protocol, regardless of demographic factors. This finding further supports the safe feasibility of using these approaches, including VR technology, to educate patients about hypertension.

## 4. Discussion

This randomised controlled trial demonstrated a significant 4-point median score advantage for VR-based education over traditional physician-led education in improving hypertension knowledge. The VR group achieved a median score of 14 compared with 10 in the control group. This robust difference highlights the significant potential of immersive VR as a powerful educational innovation for conditions that require patient understanding, such as hypertension [10,11].

While VR objectively outperformed the traditional approach for knowledge acquisition, both modalities received high satisfaction ratings from over 80% of participants. This divergence underlines that human connection remains an integral part of holistic, patient-centered education. VR should complement, not replace, face-to-face education [13,14].

The significant improvement in knowledge retention through VR-based education observed in our study is echoed in a variety of contexts in the current literature. One study demonstrated the effective use of VR to educate patients about atrial fibrillation and stroke prevention, leading to a significant improvement in patient understanding over time [23]. In the context of structural heart disease, another study found that VR experiences significantly increased patient satisfaction and understanding of their condition, which correlated with higher procedural conversion rates [4].

Consistent with our findings on patient satisfaction, other research has shown that VR can help educate patients about atrial fibrillation ablation procedures, increasing satisfaction and self-efficacy [24]. Another study reported that VR improved the understanding of patients considering treatment for movement disorders, suggesting that VR can greatly aid patient understanding in complex medical scenarios [25]. Research from China showed that VR education about radiotherapy led to improved understanding and reduced patient anxiety [26], and a study from South Korea showed that VR could reduce stress and anxiety in paediatric patients undergoing chest radiography, potentially improving the radiographic process [27].

Taken together, these studies highlight the multiple benefits of VR in patient education, going beyond knowledge enhancement to include patient satisfaction and emotional well-being. They support the integration of VR into a wide range of educational strategies in healthcare, underlining its effectiveness in providing deeper understanding and more engaging learning experiences for patients with complex medical conditions.

The discrepancy between VR’s superior objective outcomes and equivalent satisfaction underscores the importance of multidimensional measures in educational research. Relying solely on subjective measures may miss important differences. This finding provides valuable insight into the complex nature of patient satisfaction. [15,16].

Several factors may explain the knowledge gains of VR. The immersive and interactive properties of VR may promote greater engagement, conceptual understanding, and relatability compared to passive techniques [17,18]. Presenting information in customisable 3D environments tailored to learners’ needs may also optimize understanding [19,20]. However, human interaction remains essential for building rapport [21,22].

Limitations of this single-centre study include limited generalisability due to the context of the study and the potential impact of patients’ educational background, which was not assessed [27]. Patients’ willingness and ability to acquire knowledge, which could influence the effectiveness of the educational interventions, was also not explicitly measured. In addition, the time available for each educational session and the variability in the delivery of information by physicians may have influenced the results, although efforts were made to standardise these factors. These variables, together with potential sample size and power limitations, lack of baseline knowledge assessment and short follow-up, are recognised as limitations [28,29,30]. Self-report measures of subjective satisfaction and the unvalidated knowledge tool may be subject to inherent bias [27,28].

Accessibility barriers such as cost, technical skills, and cyber-sickness should be considered before widespread implementation, especially in vulnerable populations [30,31,32]. However, there were no adverse effects, demonstrating feasibility.

VR education may be beneficial for other complex conditions beyond hypertension [33]. However, further research is needed to confirm generalisability, assess retention, and design optimal customisable content.

Overall, this rigorous randomised trial provides strong initial evidence to support the integration of VR into hypertension education to improve patient knowledge and empower self-management [7,34,35].

## 5. Conclusions

In conclusion, this randomised controlled trial provides initial evidence to support virtual reality as an effective educational innovation for patients with hypertension, demonstrating an objective advantage in knowledge retention over traditional physician-led education. The significant 4-point difference in median scores highlights the promise of VR and supports its integration into hypertension education programmes. The immersive properties of VR appear to facilitate improved comprehension of self-management concepts that require patient understanding. However, human interaction remains essential; VR should complement, not replace, traditional techniques and the doctor–patient relationship. The discrepancy between knowledge gains and satisfaction highlights the need for multidimensional evaluations in educational research, as reliance on subjective measures alone may overlook objective effects. Further high-quality studies confirming generalisability, investigating sustainability, and tailoring content will help to refine the optimal integration of VR into best-practice hypertension education.

## Figures and Tables

**Table 1:** Baseline Characteristics of the Patients.

Characteristics			Education Method		
		Total Cohort	Physician-Led Education	VR Education	Stats (*p*=)
Sex	total (N)	182	88	94	
	female (N; %)	83 (46%)	40 (45%)	43 (46%)	0.969
	male (N; %)	99 (54%)	48 (55%)	51 (54%)	
Age (years) (median; IQR)		66 (16)	66 (16)	66 (17)	0.448
Systolic blood pressure (mmHg) (median; IQR)		131 (17)	132 (20)	130 (15)	0.716
Diastolic blood pressure (mmHg) (median; IQR)		80 (10)	80 (10)	80 (9)	0.797
Number of antihypertensive drugs (median; IQR)		2 (1)	2 (1)	2 (1)	0.091
Optimal Home Monitored BP Control (N; %)		137 (76.1%)	72 (81.8%)	65 (70.7%)	0.079
Hypertension-related Complications (N; %)		120 (66.7%)	58 (65.9%)	62 (67.4%)	0.833
Diabetes Mellitus (N; %)		55 (30.6%)	31 (35.2%)	24 (26.1%)	0.183
Dyslipidemia (N; %)		150 (83.3%)	76 (86.4%)	74 (80.4%)	0.286

This table presents the baseline characteristics of participants, both for the entire cohort and stratified by the education method: traditional physician-led education or Virtual Reality (VR) education. Categorical variables, such as Sex, Optimal Home Monitored BP Control, Hypertension-related Complications, Diabetes Mellitus, and Dyslipidemia, are presented as number (N) and percentage (%). Continuous variables, such as age, systolic and diastolic blood pressure, and number of antihypertensive drugs, are presented as median values with their interquartile range (IQR). Differences between groups were assessed using the Pearson chi-square test for categorical variables and the Mann–Whitney U test for continuous variables with non-normal distributions. A *p*-value less than 0.05 indicates statistical significance. All *p*-values are two-sided.

**Table 2:** Knowledge by Education Method.

Knowledge Assessment			Education Method				
	Total		Physician		VR		Stats
	N	Median (IQR)	N	Median (IQR)	N	Median (IQR)	*p*=
Total Objective Score	182	12 (4)	88	10 (5)	94	14 (3)	<0.001

This table displays Knowledge by Education Method by presenting the total objective scores of patients in both the physician and VR groups. The total objective score was calculated by adding 1 point for each correct answer and subtracting 1 point for each incorrect answer in six multivariate questions, with a possible maximum of 29 total points. Scores are represented as median with the interquartile range (IQR). Comparisons were made using the Mann–Whitney U test due to non-normal distribution. A *p*-value less than 0.05 is considered statistically significant. All *p*-values are two-sided.

**Table 3:** Patients’ Subjective Satisfaction of the Education Method.

Satisfaction Level			Education Method		
		Total	Physician	VR	Stats (*p*=)
		N	N (%)	N (%)	
Subjective Score	Total	182	88	94	
	1	153	71 (81%)	82 (87%)	0.535
	2	23	13 (15%)	10 (11%)	
	3	5	3 (3%)	2 (2%)	
	4	1	1 (1%)	0	
	5	0	0	0	
	mean (SD)		1.25 (0.61)	1.15 (0.43)	

This table displays the subjective evaluations of the education method, given by patients in the physician and VR groups. Patients used an ordinal scale, with 1 being the best and 5 being the worst. The values are given as number (N) and column percentage (%). Comparisons were made using the Pearson chi-square test. Additionally, for simplicity, the subjective scores are treated as continuous variables, with values expressed as mean (standard deviation, SD). A *p*-value less than 0.05 is considered statistically significant. All *p*-values are two-sided.

**Table 4:** Comparison of Low versus Med-High Total Score Responders.

Responder Characteristics			Low versus Med-High Total Score Responders		
		Total	Low Responders (Q1 in Each Education Group)	Med-High Responders ((Q2–Q4) in Each Education Group)	Stats (*p*=)
Sex (N;%)	female	83 (46%)	35 (57%)	48 (40%)	0.024
	male	99 (54%)	26 (43%)	73 (60%)	
Age (years) (median; IQR)		66 (16)	68 (16)	65 (16)	0.036
Subjective Score (median; IQR)		1 (0)	1 (0)	1 (0)	0.295

This table contrasts the characteristics of low responders (those who scored in the first quartile (Q1) of total objective scores in each education group) and med-high responders (those who scored in the second to fourth quartiles (Q2–Q4) of total objective scores in each education group). The characteristics compared include sex, age, and subjective score. For each characteristic, the total number (N) and percentage (%) or median and interquartile range (IQR) are shown for the total cohort, as well as separately for the low and med-high responders. The *p*-values in the last column were obtained from Pearson chi-square tests for categorical variables (sex) and Mann–Whitney U tests for continuous variables with non-normal distribution (age and subjective score). Footnote: A *p*-value less than 0.05 is taken as statistically significant. Older women tended to be more prevalent in the low responder group, with statistically significant differences observed in sex and age between low and med-high responders. There was no statistically significant difference in the subjective scores between the two groups, suggesting that the level of liking for the education method did not necessarily correlate with the objective score outcomes.

## Data Availability

Data is available on request.

## Supplementary Materials

The following supporting information can be downloaded at: https://www.mdpi.com/article/10.3390/jcdd10120481/s1, Table S1. Measures of Dispersion for Total Objective Scores; Table S2. Levene’s Test of Homogeneity of Variances for Total Objective Scores; Table S3. Subanalysis of Objective Test Results by Question and Education Method.
