# Supplementary material for: Virtual Reality for Patient Education about Hypertension: A Randomized Pilot Study
Source: J Cardiovasc Dev Dis. 2023 Nov 29;10(12):481. doi: 10.3390/jcdd10120481 (PMC10744175; doi:10.3390/jcdd10120481)
Supplement: Supplementary file 1 [file jcdd-10-00481-s001.zip › jcdd-2716368-supplementary.pdf]

List of supplementary tables:

Supplementary Table S1. Measures of Dispersion for Total Objective Scores
Supplementary Table S2. Levene's Test of Homogeneity of Variances for Total Objective Scores
Supplementary Table S3. Subanalysis of Objective Test Results by Question and Education Method

Supplementary Table S1. Measures of Dispersion for Total Objective Scores.

		Total	Education method	
			Physician	VR
Total Objective Score	Standard Deviation	3.6	3.5	2.8
	Interquartile Range	4.0	5.5	3.0
	Variance	13.1	12.6	7.7

This table presents the measures of dispersion (standard deviation, interquartile range, and variance) for the total objective scores across the total cohort and then separately for the two education methods (Physician and VR). Lower values in the VR group across all three measures suggest more homogeneous (consistent) results compared to the Physician-based education method.

Supplementary Table S2. Levene's Test of Homogeneity of Variances for Total Objective Scores.

		Levene Statistic	df1	df2	Sig.
Total Objective Score	Based on Mean	9.025	1	180	0.003
	Based on Median	6.064	1	180	0.015
	Based on Median and with adjusted df	6.064	1	179.949	0.015
	Based on trimmed mean	9.328	1	180	0.003

This table presents the results of Levene's test, examining the equality of variances for total objective scores between the two education methods (Physician and VR). The test was conducted using different measures of central tendency: mean, median, median with adjusted degrees of freedom (df), and trimmed mean. The Levene statistic, degrees of freedom (df1 and df2), and significance level (Sig.) are reported for each test.

Supplementary Table S3. Subanalysis of Objective Test Results by Question and Education Method.

Arterial hypertension …	Question	Total	Education method		stats
			Physician	VR	
		Column N %	Column N %	Column N %	p=
Definition	1A	6.6%	10.2%	3.2%	0.056
	1B	10.4%	13.6%	7.4%	0.172
	1C	5.5%	4.5%	6.4%	0.587
	1D	13.2%	17.0%	9.6%	0.137
Risk factors	2A	18.1%	34.1%	3.2%	<0.001
	2B	9.3%	11.4%	7.4%	0.364
	2C	9.9%	15.9%	4.3%	0.008
	2D	17.0%	23.9%	10.6%	0.018
	2E	40.7%	51.1%	30.9%	0.005
	2F	17.0%	29.5%	5.3%	<0.001
	2G	18.7%	29.5%	8.5%	<0.001
Symptoms	3A	25.3%	44.3%	7.4%	<0.001
	3B	40.1%	46.6%	34.0%	0.084
	3C	45.1%	76.1%	16.0%	<0.001
	3D	36.3%	39.8%	33.0%	0.341
	3E	4.4%	5.7%	3.2%	0.413
Regular medication use	4A	7.7%	9.1%	6.4%	0.493
	4B	0.5%	0.0%	1.1%	0.332
	4C	3.3%	3.4%	3.2%	0.935
	4D	0.0%	0.0%	0.0%	NA
Home blood preasure measurement	5A	9.3%	12.5%	6.4%	0.156
	5B	0.5%	1.1%	0.0%	0.3
	5C	26.9%	43.2%	11.7%	<0.001
	5D	44.5%	62.5%	27.7%	<0.001
	5E	3.8%	6.8%	1.1%	0.044
Complications	6A	4.9%	6.8%	3.2%	0.259
	6B	2.7%	2.3%	3.2%	0.705
	6C	44.5%	64.8%	25.5%	<0.001
	6D	15.4%	23.9%	7.4%	0.002

This table presents the subanalysis of the objective test results by each question and the method of education, either by a Physician or using Virtual Reality (VR). The table is organized by sections, with each section corresponding to a specific domain of arterial hypertension knowledge: Definition, Risk Factors, Symptoms,

Regular Medication Use, Home Blood Pressure Measurement, and Complications. For each question within these domains, the frequency (percentage) of incorrect answers is reported, separately for the Physician and VR groups, as well as for the total cohort. The p-values in the final column were calculated using Pearson chi-square tests, comparing the frequency of incorrect answers between the Physician and VR groups. Footnote: The p-value indicates the level of significance for the differences between the VR and Physician groups in terms of the proportion of incorrect answers for each question. P-values less than 0.05 were considered statistically significant. This subanalysis demonstrates specific areas where the VR method of education resulted in fewer incorrect answers, suggesting possible advantages of VR education in these areas. Note: NA (not applicable) is listed for question 4D due to zero incorrect answers in both groups.
